# Photocatalytic removal of 2-chlorophenol from water by using waste eggshell-derived calcium ferrite

**DOI:** 10.1039/d3ra01357j

**Published:** 2023-06-12

**Authors:** Suwilai Chaveanghong, Thawanrat Kobkeatthawin, Jirawat Trakulmututa, Taweechai Amornsakchai, Puangrat Kajitvichyanukul, Siwaporn Meejoo Smith

**Affiliations:** a Center of Sustainable Energy and Green Materials and Department of Chemistry, Faculty of Science, Mahidol University 999 Phuttamonthon Sai 4 Rd, Salaya Nakhon Pathom 73170 Thailand suwilai.cha@mahidol.ac.th kunthidakob@gmail.com jirawat.trk@student.mahidol.edu tawee-chai.amo@mahidol.ac.th siwaporn.smi@mahidol.edu; b Mahidol University Frontier Research Facility, Mahidol University 999 Phuttamonthon Sai 4 Rd, Salaya Nakhon Pathom 73170 Thailand suwilai.cha@mahidol.ac.th; c Department of Environmental Engineering, Faculty of Engineering, Chiang Mai University 239, Huay Kaew Road, Muang District Chiang Mai 50200 Thailand puangrat.k@cmu.ac.th; d Sustainable Engineering Research Center for Pollution and Environmental Management, Faculty of Engineering, Chiang Mai University 239, Huay Kaew Road, Muang District Chiang Mai 50200 Thailand

## Abstract

A new approach to recycling low-value eggshell food waste was to produce a CaFe_2_O_4_ semiconductor with a narrow band gap (*E*_g_ = 2.81 eV) *via* hydrothermal treatments of powdered eggshell suspended in aqueous ferric salt (Fe^3+^) solutions at varying Fe loadings. It was possible to obtain a single phase of CaFe_2_O_4_ without any Ca(OH)_2_ and CaO impurities using an optimal Fe loading (30 wt% of Fe^3+^ by eggshell weight). The CaFe_2_O_4_ material was used as a photocatalyst for the breakdown of 2-chlorophenol (2-CP, a herbicide model chemical) as a pollutant in water. The CaFe_2_O_4_ with a Fe loading of 7.1 wt% exhibited a high 2-CP removal efficiency of 86.1% after 180 min of UV-visible light irradiation. Additionally, the eggshell-derived CaFe_2_O_4_ photocatalyst can be effectively reused, giving a high removal efficiency of 70.5% after the third cycle, without the requirement of regeneration processes (washing or re-calcination). Although radical trapping experiments confirmed that hydroxyl radicals were generated in the photocatalytic reactions, photogenerated holes play a significant role in the high 2-CP degradation efficiencies. The performance of the bioderived CaFe_2_O_4_ photocatalysts in the removal of pesticides from water demonstrated the benefits of resource recycling in the area of materials science and in environmental remediation and protection.

## Abstract 2

A new approach to recycling low-value eggshell food waste was to produce a CaFe_2_O_4_ semiconductor with a narrow band gap (*E*_g_ = 2.81 eV) *via* hydrothermal treatments of powdered eggshell suspended in aqueous ferric salt (Fe^3+^) solutions at varying Fe loadings.

## Graphical abstract

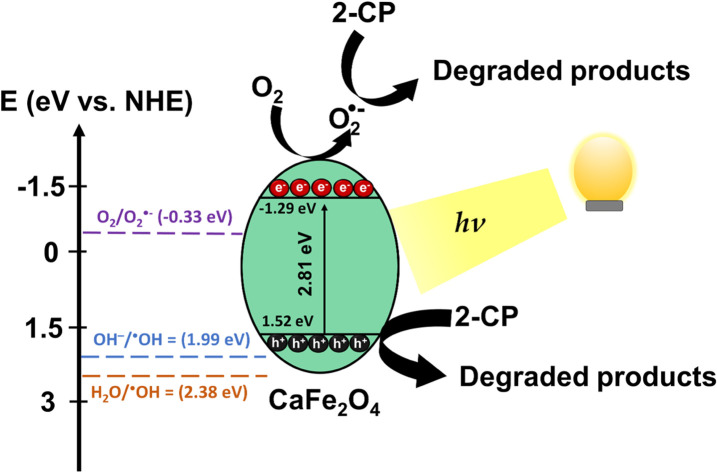

## Introduction

Chlorophenol is one of the most persistent herbicides, and according to the Environment Protection Agency (EPA), 2-chlorophenol (2-CP) is one of the environmental priority pollutants due to its high toxicity and poor biodegradation. This is a result of the high stability of the C–Cl bond in the 2-CP structure.^1–3^ As industrialized farming needs to ensure high yields of excellent quality, *i.e.*, good-looking fruits or vegetables free from insect damage, many farmers apply a large amount of pesticides in their farmlands. Typically, the run-off containing residual pesticides results in water pollution and the pesticides enter the food-chain of aquatic plants and animals. Adsorption is one promising method for the removal of chlorophenol in contaminated water. However, the adsorption method required a relatively high adsorbent dosage (1 g L^−1^ to 25 g L^−1^) and further processes to degrade such pollutants.^4–6^ Alternatively, photocatalysis technology has received considerable attention owing to its outstanding performance in the degradation of a wide range of toxic and non-degradable pollutants in water, converting them to less-toxic molecules and/or carbon dioxide. The photocatalytic wastewater treatments produce no sludge waste, hence being potentially cost effective.^7–10^ Photocatalytic ozonation was reported as a superior effective method to ozonation and Fenton oxidation in the removal of dichlorophenol in a dielectric barrier discharge under varying gas atmospheres.^11,12^ Ozone is added into the reactor as an oxidant to enhance the dichlorophenol degradation processes. In many reports, several synthesized photocatalysts, *e.g.*, chemically modified TiO_2_, CuInS_2_/TiO_2_ and TiO_2_/AgInS_2_ heterojunctions, were applied in the degradation of chlorophenols without the requirement of any oxidant addition.^13–16^ However, the reported photocatalysts have some drawbacks, requiring expensive and high-purity precursors in multi-step preparation processes.

As a semiconductor, calcium ferrite (CaFe_2_O_4_) has been extensively applied as a photoactive material for many applications, such as CO_2_ conversion,^17^ solar water oxidation,^18^ hydrogen production,^19,20^ ferrite pigments^21^ and photocatalyst for water depollution.^22^ The co-precipitation method is a simple and cost-effective route to produce calcium ferrites, while calcium and ferric (ferrous) salts are commonly utilized as precursors.^22–24^ Since mining for finite resources may become more expensive and impractical in the future, wastes or renewable raw materials have also gained popularity due to their sustainable nature and potential to reduce carbon emissions. It has been reported that a spinel calcium ferrite can be prepared from steel industry waste and from the recycling of electric arc furnace dust.^25^ This innovative approach not only reduces waste, but also creates value of such waste in the area of photocatalyst development.

Eggshells, a biobased calcium source, are potentially renewable feedstocks for the materials development industry, serving as an alternative to high-purity chemicals or finite limestone. Eggshell food waste, containing CaCO_3_ as the main composition, can be sorted, recovered and utilized in industries, such as fertilizer, animal feed, cement tile, sorbents, and biodiesel production plants.^26^ One of the reports given by the WATT Global Media's Executive Guide to World Poultry Trends suggested a large quantity of eggshell waste, around 8.58 million metric tons of worldwide, in 2018.^26^ Calcinations of eggshell produced CaO, which is a common raw material in glass, cement, paper, and high-grade steel plants. To use bioresources (or wastes) in material synthesis, it is important to determine the optimum process to produce a high-value material with high purity. This report aims to propose a green synthesis approach using waste eggshells (abundant, low-value, and renewable resources) as a calcium precursor in CaFe_2_O_4_ production. In addition, the photocatalytic performance of CaFe_2_O_4_ materials in the treatment of aqueous 2-cholophenol (a biocide and antiseptic substance) was investigated, exploring the photocatalytic activity, including the determination of key factors influencing the photocatalytic activity of bio-waste derived materials. This work should broaden insight into the relationships among the composition, physiochemical (including optical) properties, and the photocatalytic activity of the materials. The results and key findings from this research may also promote sustainable food waste utilization *via* creating an additional application of eggshell waste as a raw material in the production of high-value semiconducting materials being used in various applications not only for the remediation of agricultural wastewater, but also photoactive, magnetic and drug delivery applications.^27,28^ Additionally, the use of renewable raw materials can contribute to the development of a circular economy by reducing waste and promoting resource efficiency.

## Experimental

### Materials

Quail eggshell (QES) waste collected from the local market and iron(iii) chloride (FeCl_3_, AR grade, Sigma-Aldrich) were used as the Fe precursor. 2-Chlorophenol (2-CP, AR grade, Merck), ammonium oxalate (AR grade, Merck), benzoquinone (AR grade, Sigma-Aldrich) and isopropyl alcohol (AR grade, QRëc) were used as purchased.

### Photocatalyst preparation

Calcium ferrite, a QES-based photocatalyst, was prepared by using the modified method reported by our group.^29^ Firstly, dried powdered QES was sieved (400 mesh) to obtain particles with the particle sizes lower than 37 μm. Calcination of QES at 750 °C for 6 h resulted in CaO. Secondly, FeCl_3_ (3 g) dissolved in DI water (200 mL) was mixed with QES (7 g). Next, the suspension was sonicated for 30 min. Thereafter, the mixture was transferred to a Teflon-lined stainless steel reactor for hydrothermal treatment (120 °C, 4 h), followed by calcination at 750 °C for 6 h. It should be noted that 10Fe/QES and 20Fe/QES were prepared with similar preparation procedures, varying the Fe loadings (10–20 wt% with respect to that of QES). The calcinations resulted in *ca.* 50% mass loss of the uncalcined samples due to the conversion of CaCO_3_ to CO_2_.

### Photocatalyst characterizations

Phase identification of the powdered samples was performed by Powder X-ray Diffraction (PXRD) technique on a Bruker AXS model D8 Advance diffractometer equipped with Cu Kα radiation (*λ* = 1.5418 Å), 40 mA and 40 kV, 2*θ* range = 5–80°, step = 0.075°, scan step = 0.2° s^−1^. The crystalline phases of all samples were identified based on reference data from the Joint Committee on Powder Diffraction Standards (JCPDS) database. Microstructural studies of the catalysts were performed on a scanning electron microscope (SEM; Hitachi: SU8010). The samples were coated with platinum prior to study by SEM. The surface chemical composition/species of the samples was studied using X-ray photoelectron spectroscopy (XPS; Kratos Model Axis ultra DLD). The samples are excited with X-ray hybrid mode 700 × 300 μm spot area with a monochromatic Al Kα_1,2_ radiation at 1.4 keV. All spectra are calibrated using the C 1s line (binding energy; BE = 285.0 eV). The optical band gap energy of the prepared samples was observed by UV-vis diffuse reflectance spectroscopy (DRS, Shimadzu, UV-2600) with BaSO_4_ as a reference. FTIR-ATR spectra were measured on a spectrophotometer (Thermo Scientific, Nicolet 6700) within the range of 4000 to 400 cm^−1^, using 16 scans of accumulations at 4 cm^−1^ of resolution. X-ray fluorescence spectroscopy (XRF, Horiba, XGT-9000) was employed to measure the bulk concentration of elements in the samples. ESR spectra were measured at 25 °C, using a power of 20.02 mW and a frequency of 9.84 GHz on a Bruker Elexys E500 cw X-band ESR spectrometer equipped with an Oxford ITC605 temperature controller.

### Photocatalytic activity screening test and reusability

In the photocatalytic degradation batch testing, the as-prepared photocatalyst powder (2.5 g L^−1^) was firstly suspended under magnetic stirring in synthetic wastewater containing 2-chlorophenol (2-CP, 25 ppm, 10 mL) in the dark for 30 min to reach adsorption–desorption equilibrium. When *x*Fe/QES (*x* = % Fe loading) was added to 2-CP (aq), the pH of the solution increased from 6.0 to 12. Then, each suspension was illuminated (UV-vis radiation, OSRAM Ultra-vitalux 300 W × 2; *λ* > 280 nm) at different reaction times (30, 60, 120, and 180 min), at a temperature of 35.0 ± 3.0 °C.^30^ A syringe filter (0.45 μm nylon filter membrane) was used to withdraw the treated 2-CP(aq) from each batch reactor for UV-vis absorption spectrum measurement (GENESYS 10s UV-Vis spectrophotometer). The concentration of 2-CP was monitored by measuring the absorbance at *λ* = 237 nm,^31–33^ following the protocol reported previously.^34–36^ The % photocatalytic degradation efficiencies were calculated using the formula below.

1 % Photocatalytic degradation efficiency = [(*C*_0_ − *C*_*t*_)/*C*_0_] × 100

where *C*_0_ is the initial concentration of the pollutant model (25 ppm) and *C*_*t*_ is the concentration of the pollutant model after treatment after specific treatment times. To confirm the 2-CP degradation, the total organic carbon (TOC) values were measured for the treated 2-CP(aq). The TOC values of the treated 2-CP (aq) solutions were determined on a TOC analyzer (analyticjena, Multi N/C 2100 S). Then, the TOC removal efficiencies were calculated as follows.

2 % TOC removal efficiency = [(TOC_0_ − TOC_*t*_)/TOC_0_] × 100

where TOC_0_ is the initial TOC value of the 25 ppm 2-CP (aq), and TOC_*t*_ is the TOC content after treatment at particular treatment periods.

Furthermore, reusability tests were carried out while the treated 2-CP (aq) was decanted after centrifugation, and fresh 25 ppm 2-CP (aq) was added in the batch reactor to examine the photocatalytic activity of the spent catalyst in subsequent runs (180 min treatments). Notably, the spent photocatalyst was reused in the next run without washing or any regeneration.

### Degradation product determination

Gas chromatography-mass spectrometry (GC-MS, Agilent Technologies 5977B MSD) was employed to identify any 2-CP degradation products in the treated aqueous 2-CP, using the previously reported protocol with small adjustment.^3,37^ The GC-MS measurements were performed by using a DB-5MS column, having dimensions of 30 m × 0.25 mm × 0.25 μm with the injection volume of the sample at 1 μL (flow 1.0 mL min^−1^). The controlled oven temperature program included maintaining at 60 °C for 1 min, and increasing by 20 °C min^−1^ up to 300 °C.

### Probing of reactive oxygen radicals (OH˙ and O_2_˙^−^) and holes (h^+^)

Semi-quantitative analysis of reactive oxygen species (such as OH˙ and O_2_˙^−^ radicals) and photogenerated holes in the studied photocatalysis system was carried out by electron spin resonance (ESR) techniques using aqueous 5,5,-dimethylpyrroline N-oxide (DMPO, TCI chemical purity >97%) spin trapping.^38–40^ The as-prepared catalyst (10 mg) was suspended in a 2-CP aqueous solution (25 ppm, 4 mL) under magnetic stirring for 30 min in the dark. Subsequently, aqueous DMPO (50 mmol L^−1^, 1 mL) was added in the suspension for 2 min well mixing, followed by filtering using a 0.45 μM syringe filter. The ESR spectrum of the filtered sample was measured using the same protocol as that of the aqueous 2-CP solution after adsorption treatments. On the other hand, the suspensions of catalyst sample in DMPO/2-CP (aq) that underwent adsorption treatment were subjected to UV-vis light irradiation (OSRAM Ultra-vitalux 300 W × 2; *λ* > 280 nm as a light source) at given time intervals (15, 30 and 60 min), followed by ESR measurements. In the case of O_2_˙^−^ probing, an identical procedure was carried out. However, methanolic DMPO was used to enhance the ESR signals. For comparison, QES-750 and Fe loaded QES-750 underwent similar tests to confirm whether any reactive oxygen species were generated by the ferrite precursor materials.

### Scavenger tests

The scavenging test was carried out following the protocol previously reported.^41,42^ Three scavenging agents, ammonium oxalate (AO), benzoquinone (BQ) and isopropyl alcohol (IPA), were each added into 2-CP (aq) at the 2-CP-to-scavenger molar ratio of 1 : 2, to quantify the amount of photogenerated holes, O_2_˙^−^ and OH˙ radicals, respectively. Despite being a reducible organic compound, BQ is sufficiently effective to trap holes, and suppressed the 2-CP degradation rate at pH 12. The intensity of the absorption peak at *λ* = 237 nm does not overlap with any other peaks that correspond to other degradation products and BQ. The lower 2-CP removal efficiencies obtained in the scavenging agent-containing systems reflected the effective scavenging reactions.^41,42^

## Results and discussion

### Materials characterization

**Fig. 1:**
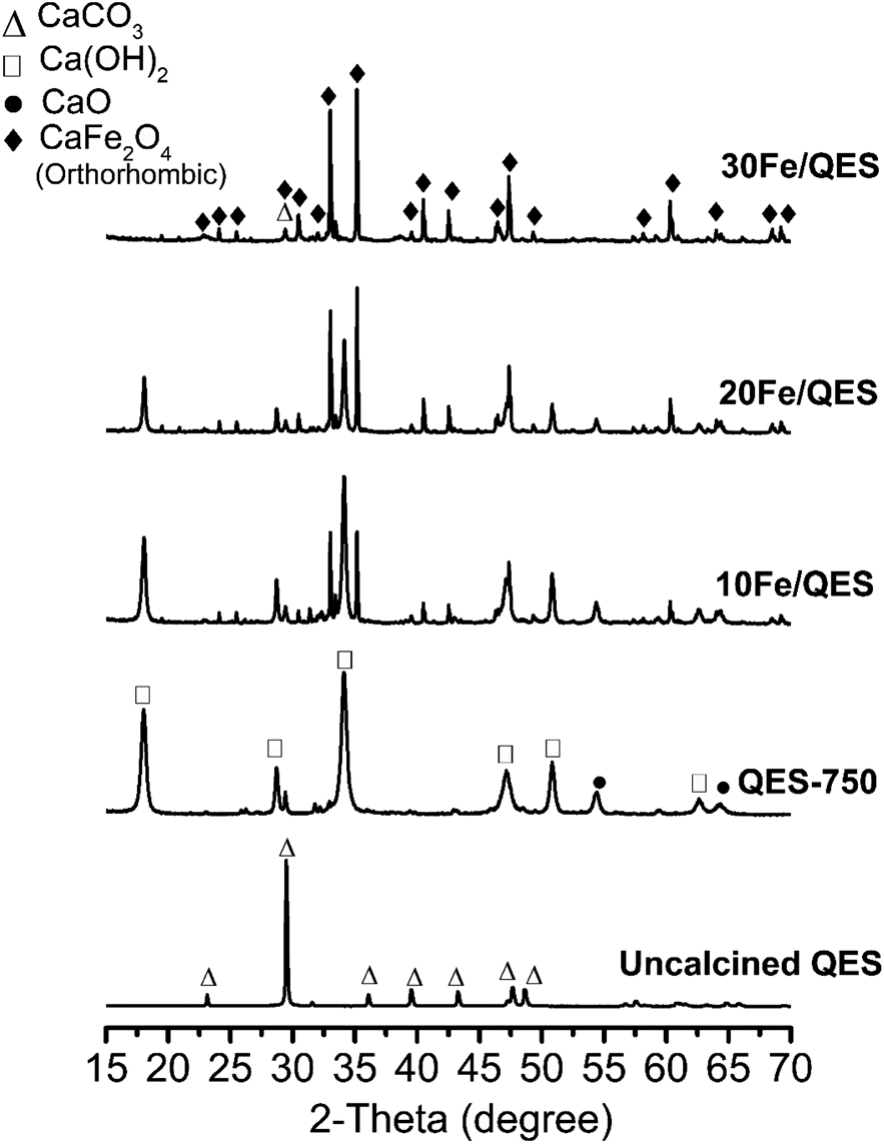
PXRD of uncalcined QES, QES and Fe-loaded QES after calcination at 750 °C for 6 h.

Powder X-ray diffraction (PXRD) patterns for the Fe-loaded quail eggshell (10–30% wt Fe/QES) after calcination are shown in Fig. 1. Notably, uncalcined eggshell was denoted as QES (or uncalcined QES), whereas the calcined eggshell was denoted as QES-750. The calcined Fe-loaded eggshell is denoted as *x*Fe/QES, while *x* = % FeCl_3_ loading with respect to the eggshell weight. It was observed that uncalcined QES mainly consisted of crystalline calcium carbonate (CaCO_3_), showing a major peak at 2*θ* = 29.5°.^43^ After calcination at 750 °C, decomposition of CaCO_3_ in the QES resulted in the formation of CaO and Ca(OH)_2_ (or hydrated CaO), resulting from CaCO_3_ decomposition, in excellent agreement with previous studies.^43,44^ With Fe loading, the 10Fe/QES and 20Fe/QES samples contained a mixture of Ca(OH)_2_, CaO and CaFe_2_O_4_.^45^ Based on the diffraction profiles, only the 30Fe/QES sample comprises CaFe_2_O_4_, along with unreacted CaCO_3_ as the minor phase (2*θ* = 29.5°). Hence, 30% FeCl_3_ loading is the optimum condition in the preparation of the highest purity of CaFe_2_O_4_. The amount of CaO phase (if present) in the 30Fe/QES sample is lower than the detection limit of powder diffraction techniques.

**Fig. 2:**
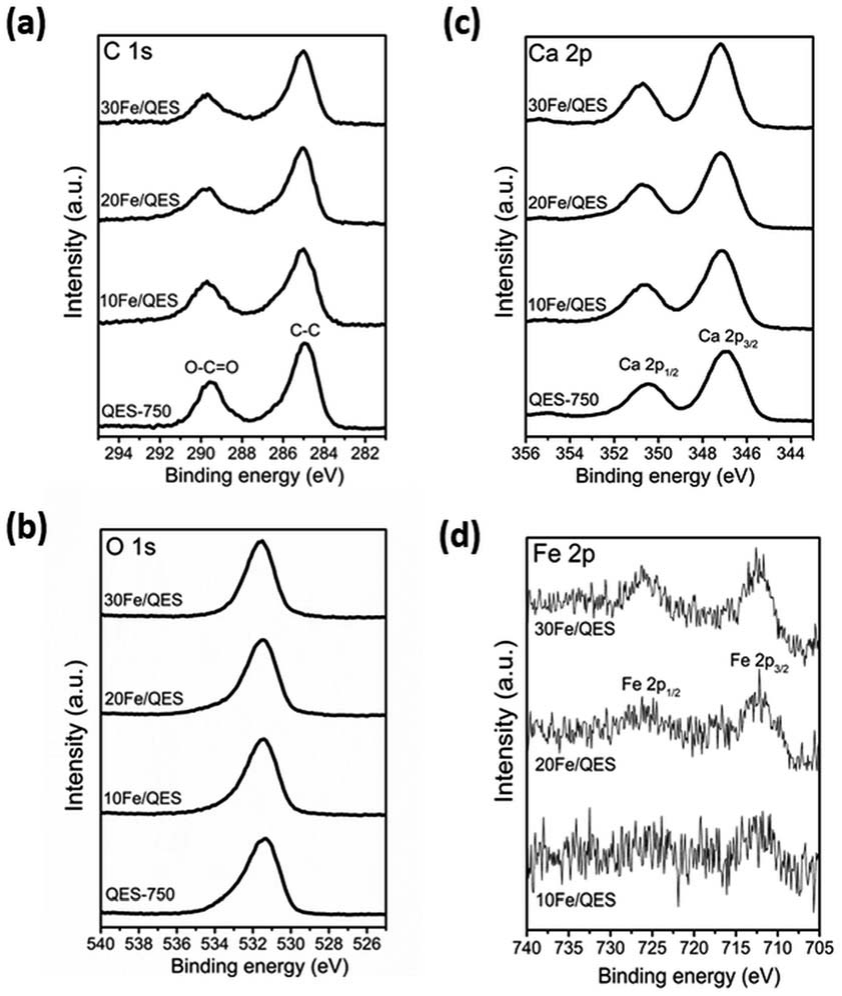
High-resolution XPS spectra of calcined QES and Fe-loaded QES after calcination (a) C 1s, (b) O 1s, (c) Ca 2p and (d) Fe 2p.

**Fig. 3:**
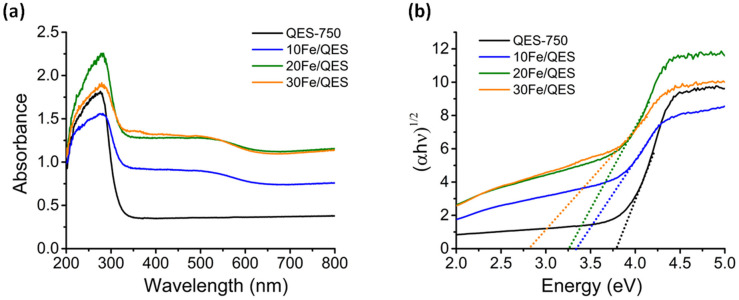
(a) UV-vis diffuse reflectance spectra and (b) plots of the transformed Tauc function *versus* light energy for the calcined QES and Fe-loaded QES after calcination.

Surface chemical compositions of the prepared samples were determined by XPS, and the results are shown in Fig. 2. The C 1s spectrum (Fig. 2a) of all samples shows two main peaks at 285.0 and 289.5 eV, corresponding to C–C of the surface contaminated adventitious carbon and O–C=O of the carbonate compound.^46^ The QES-750 showed a highly intense peak corresponding to the carbonate functional group (at 289.5 eV). In addition, all samples exhibited two peaks in the Ca 2p region (Fig. 2b), attributed to Ca 2p_3/2_ (347.0 eV) and Ca 2p_1/2_ (350.5 eV). In addition, O 1s (Fig. 3c) was detected in all samples at the binding energy of 531.0 eV. In Fig. 2d, all Fe loaded samples showed two deconvolution peaks at binding energies of 712. 4 eV and 726.2 eV, which corresponded to the Fe 2p_3/2_ and Fe 2p_1/2_ of CaFe_2_O_4_.^47,48^ It should be noted that the peak intensity of Fe 2p increased with increased Fe content (10 to 30 wt%), accounting for 1.8 wt% (10Fe/QES), 4.0 wt% (20Fe/QES) and 7.1 wt% (30Fe/QES). XPS is a surface technique (∼10 nm deep from the outer surface),^49,50^ and the concentration of Fe at the surface may be different from that in bulk. The catalytically active sites at the surface are important to the bulk for surface reactions, including the 2-CP degradation in this work. The high bulk concentration of Fe in 30Fe/QES is found to be 36%, which is more than the theoretical value, possibly because XRF is not suitable for the concentration determination of light elements (*i.e.*, carbon from CaCO_3_). The catalytically active sites at the surface are important for surface reactions. Notably, the XPS peaks agree well with the PXRD data, suggesting the presence of CaO, CaCO_3_ and CaFe_2_O_4_ in the 10Fe/QES and 20Fe/QES samples.

UV-vis diffuse reflectance spectroscopy (UV-DRS) was employed to investigate the light-responsive characteristics of the studied materials. In comparison with QES-750, the absorption edges of the calcined Fe-modified QES samples were found to be slightly shifted to longer wavelengths, along with a new broad absorption peak at ∼450–600 nm (Fig. 3a). Thus, the absorption spectra of the Fe-loaded QES materials indicated that they tend to be responsive to visible light. Additionally, the optical band gap energy (*E*_g_) was evaluated by extrapolating the linear portion of the curve plotted from the Tauc function *versus* light energy^51–53^ shown in Fig. 3b, exhibiting *E*_g_ values of 3.71, 3.24, 3.18, and 2.81 eV for the QES-750, 10Fe/QES, 20Fe/QES and 30Fe/QES materials, respectively. The smallest bandgap of the 30Fe/QES material is possibly due to the highest purity of CaFe_2_O_4_ (without impure CaO and Ca(OH)_2_). Nevertheless, the 30Fe/QES sample gave an *E*_g_ value that was quite high compared to that of the single phase CaFe_2_O_4_ materials reported by previous studies with the values of 1.8–1.9 eV, corresponding to the absorption edge of 688–590 nm.^52–54^ The small amount of CaCO_3_ in the 30Fe/QES sample may contribute to the relatively high *E*_g_.

**Fig. 4:**
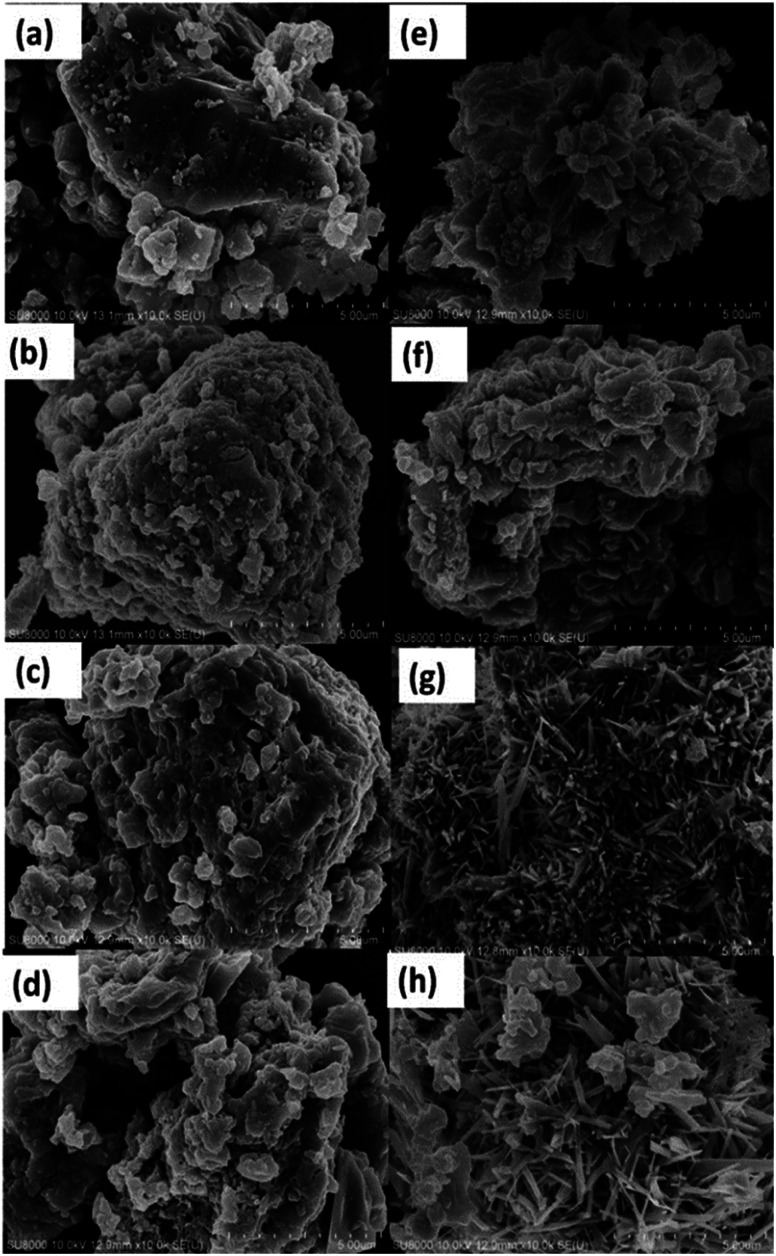
SEM images of (a) uncalcined QES, (b) uncalcined Fe-loaded QES at 10 wt%, (c) Fe-loaded QES at 20 wt% (d) Fe-loaded QES at 30 wt%, (e) QES-750, (f) 10Fe/QES, (g) 20Fe/QES, and (h) 30Fe/QES.

The influences of % Fe loading on the morphology of the *x*Fe/QES materials can be detected. The SEM image of QES eggshell particles in Fig. 4a showed various sizes of irregular granules with some pores on the surface, while the calcination, 750 °C, 6 h, of QES resulted in smaller sizes of the irregular-shaped and plate-like particles (Fig. 4e). Hydrothermal treatments of Fe-loaded QES gave irregular shaped samples having slightly irregular morphologies with rougher surface in comparison to that of QES, Fig. 4b–d. From Fig. 4f–h, the morphology of the *x*Fe/QES materials (*x* = % Fe loading) and phases (discussed earlier) depend on the % Fe loadings. Small needle-shaped particles of the 20Fe/QES and 30Fe/QES materials were observed, with 20Fe/QES having smaller and more uniform particle size.

### Photocatalytic efficiency and reusability

The *x*Fe/QES samples were applied as photocatalysts in the removal of 2-cholophenol (2-CP) from water. The use of a visible radiation light source to stimulate 2-CP breakdown was unsuccessful, giving negligible 2-CP removal efficiencies. A possible explanation for the poor photocatalytic activity under visible light irradiation is the low absorption intensity in the visible light range (Fig. 3). The aforementioned UV-vis light source was then used to explore the feasibility of the eggshell-derived photocatalysts for 2-CP degradation.

**Fig. 5:**
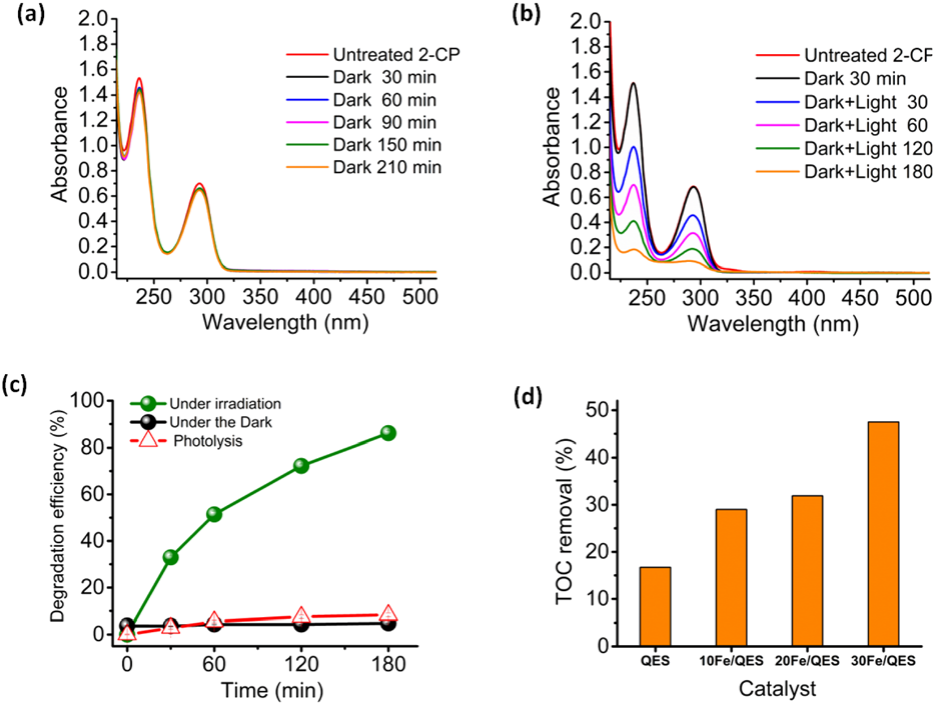
Time-dependent UV-vis spectra of 2-CP (25 ppm, pH ∼12) over 30Fe/QES with catalyst loading of 2.5 g L^−1^ (a) under dark condition and (b) under irradiation, (c) photocatalytic degradation efficiency of aqueous 2-CP (25 ppm) evaluated from the UV-vis absorption peak at 237 nm, and the (d) TOC removal efficiency (%) of 2-CP (25 ppm) over different catalysts after 180 min irradiation.

The removal of 2-CP from water was investigated under a basic condition (pH 12). No addition of base (such as NaOH) was required, as adding 30Fe/QES into 2-CP (aq) resulted in a change in pH from 6.5 to 12 due to a trace amount of the CaO phase in the 30Fe/QES sample, as suggested by XPS and XRD results. Utilizing the 30Fe/QES photocatalyst bypasses the pH adjustment step that was necessary to achieve effective degradation of 2-CP in a previous work.^35^ In addition, from a previous investigation,^55^ CaFe_2_O_4_ tends to be an ineffective photocatalyst under acidic conditions. Fig. 5a and b illustrates the absorption spectra of the aqueous 2-CP treated with 30Fe/QES. The 2-CP characteristic peaks at 237 nm and 294 nm were observed, in excellent agreement with a previous report.^2,34^ The stability of 2-CP after treatment in the dark of the 30Fe/QES sample, in Fig. 5a, suggested that there was no 2-CP removal. Hence, the photocatalytic reactions resulted in the 2-CP removal, as confirmed in Fig. 5b. From Fig. 5c, it can be seen that 8% removal of 2-CP is due to sorption (from treatment under dark condition). The highest 2-CP removal efficiency of 86.1 ± 1.0% was achieved from the 30Fe/QES treatment of 25 ppm 2-CP (aq) after 180 min UV-vis illumination.

Furthermore, the photocatalytic activity of biobased materials in the degradation of aqueous 2-CP was examined by total organic carbon (TOC) analyses. The TOC removal efficiencies after irradiation for 180 min obtained from treatments of 2-CP (aq) using various catalysts are displayed in Fig. 5d. The highest TOC removal efficiency of *ca.* 50% was achieved, upon 2-CP (aq) abatement, by using the 30Fe/QES photocatalyst. Thus, the results in Fig. 5c and d suggested a complete conversion of 2-CP to degradation products. However, the products are sufficiently stable and half of 2-CP was mineralized to CO_2_ and H_2_O. It should be noted that the bio-resourced CaO (QES-750) was found to be an ineffective photocatalyst, providing a TOC removal efficiency that was lower than 20%. The semiconducting properties of QES-750 are in a good agreement with another study that applied CaO/Ca(OH)_2_ as photocatalyst in the decomposition of organic pollutants.^55–57^

**Fig. 6:**
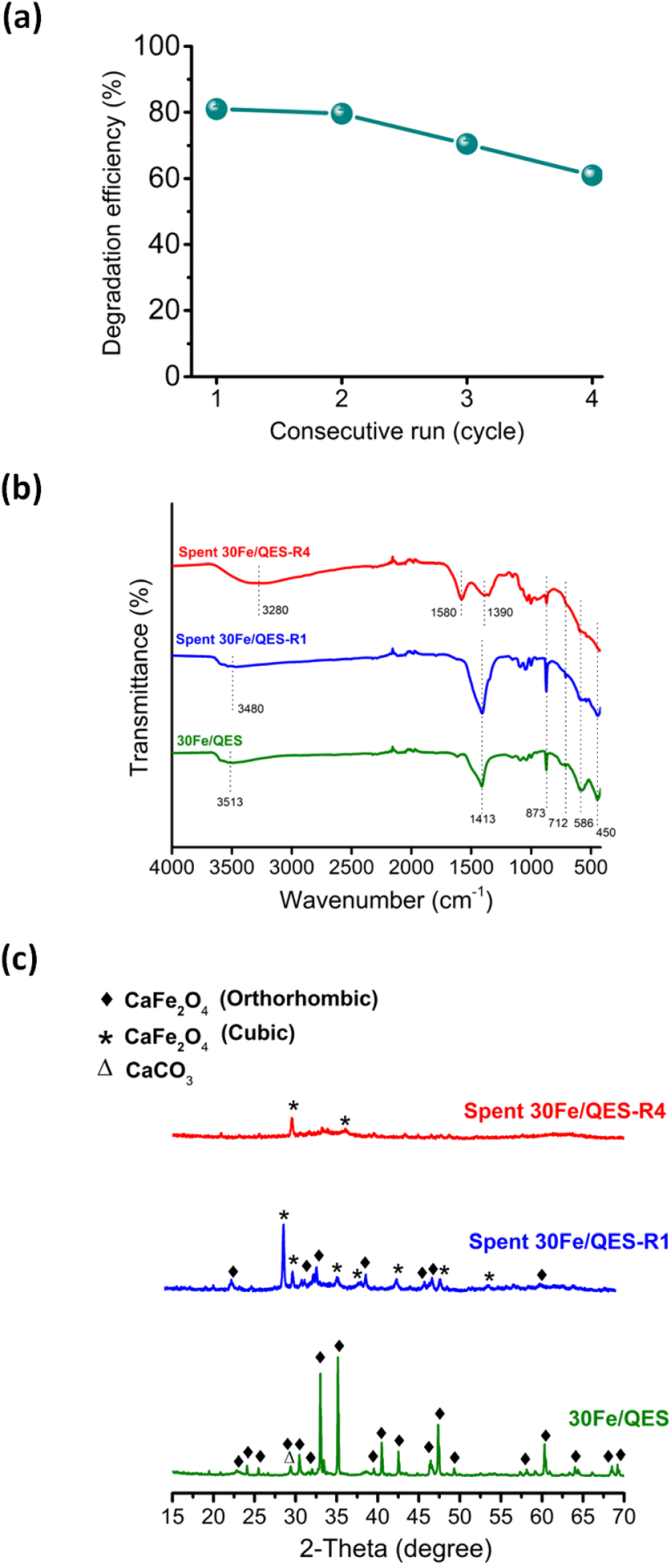
(a) 2-CP degradation in subsequent runs over 30Fe/QES (without purification/regeneration processes) under UV-vis light irradiation, (b) FT-IR spectra and (c) PXRD of 30Fe/QES before and after treatment with 2-CP (1st cycle: spent 30Fe/QES-R1, and 4th cycle: spent 30Fe/QES-R4). Prior to IR and XRD measurements, the spent catalyst was dried at 65 °C without washing.

The results shown in Fig. 5 suggested that the UV-vis absorption technique may be not suitable to monitor and identify intermediates in this reaction system. Nonetheless, the incomplete TOC removal from the 30Fe/QES treatments provides evidence of stable 2-CP degradation products in the treated 2-CP solutions. The results suggested that the 30Fe/QES photocatalyst, with a relatively low bandgap energy, may be responsible for the high 2-CP removal efficiency. Additionally, the minor CaCO_3_ phase in the 30Fe/QES photoactive material may promote the effective separation of the photogenerated electrons and holes, similar to another research work that indicated the improved photocatalytic degradation efficiencies of aqueous organic dyes (rhodamine B and methyl orange) over CaCO_3_/ZnO.^58^ Furthermore, the 30Fe/QES was subjected to subsequent 2-CP removal runs without purification/regeneration processes. It was found that the 30Fe/QES catalyst can be effectively reused, giving 2-CP removal efficiencies of 70.5% ± 0.99 in the 3rd cycle. Then, the efficiency decreased in the 4th cycle to 61.0% ± 1.98, as seen in Fig. 6d. The lower removal efficiencies may be due to the catalyst mass loss during multiple transfers of the catalyst in centrifugation and decantation steps.

From the FT-IR results (Fig. 6b), unclear absorption peaks corresponding to the CO_3_^2−^ group of CaCO_3_ at 1413, 873 and 712 cm^−1^, Fe–O (586 cm^−1^) and Ca–O (450 cm^−1^) were observed, compared to fresh 30Fe/QES and after reuse for 1 cycle.^59^ In addition, the presence of peaks in the range of 1400–1600 cm^−1^ was detected, corresponding to aromatic derivative species (degraded products). The obtained results may suggest that the surface covering 2-CP degradation products, and chlorine poisons^60^ could cause a lessening of the content of catalytic active sites on 30Fe/QES to effectively degrade 2-CP. From Fig. 6c, there is no evidence of the decomposition (photo-corrosion) of calcium ferrite upon irradiation under basic conditions. Nonetheless, ion rearrangement in calcium ferrite took place after treatments with 2-CP (aq), resulting in the structural transformation from the orthorhombic CaFe_2_O_4_ to cubic CaFe_2_O_4_ phase.^61^ The spent 30Fe/QES photocatalyst contains both polymorphs.

### Determination of the degradation products

**Fig. 7:**
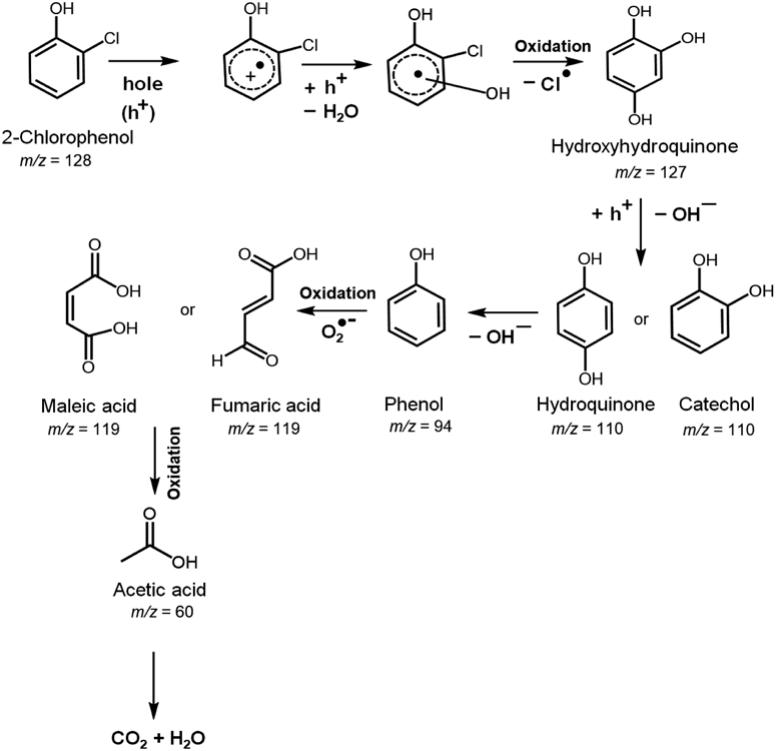
Photocatalytic degradation pathway of 2-CP over 30Fe/QES, based on GC-MS, TOC results, and previous reports.^63,64^

Fig. 7 summarizes the possible photocatalytic degradation pathway of 2-CP *via* oxidation process over 30Fe/QES after 180 min irradiation with UV-vis light. Identification of the 2-CP degradation products over 30Fe/QES was performed by GC/MS.^3,37,62^ After photodegradation for 60 min, it was found that 2-CP (*m*/*z* of 128) was degraded to hydroxy hydroquinone (*m*/*z* of 126) *via* dehalogenation (Cl group removal) and oxidation process.^37^ The loss of the OH group from hydroxyhydroquinone led to the generation of hydroquinone or catechol (*m*/*z* of 110), and further degraded to phenol (*m*/*z* of 96). Thereafter, the oxidation of phenol led to the formation of maleic acid or fumaric acid (*m*/*z* = 119). Notably, at reaction times of 120 and 180 min, the formation of acetic acid (*m*/*z* = 60) was detected, resulting from the degradation of maleic acid or fumaric acid, and further possibly degraded to smaller molecules into CO_2_ and H_2_O and HCl, as reported in previous studies.^37,62^ Results indicated that the breakdown of 2-CP requires at least 120 minutes to produce a substance that is less hazardous than 2-CP. The possible photodegradation products of 2-CP identified in Fig. 7 are quite similar to those in previous research studies.^36,62^ Nonetheless, the characteristic masses (*m*/*z*) of some intermediates were slightly different from their theoretical molecular weight (±5) probably because the compound has secondary characteristic masses or low concentration, and was converted rapidly to CO_2_ within the long reaction time.^37^

### Probing of the reactive oxygen species and holes under photocatalytic systems

ESR spectra can be indicative evidence of the photogenerated radical species in the treated 2-CP (aq). The characteristic ESR signals corresponding to the DMPO/˙OH adduct were previously reported elsewhere.^38–40^

**Fig. 8:**
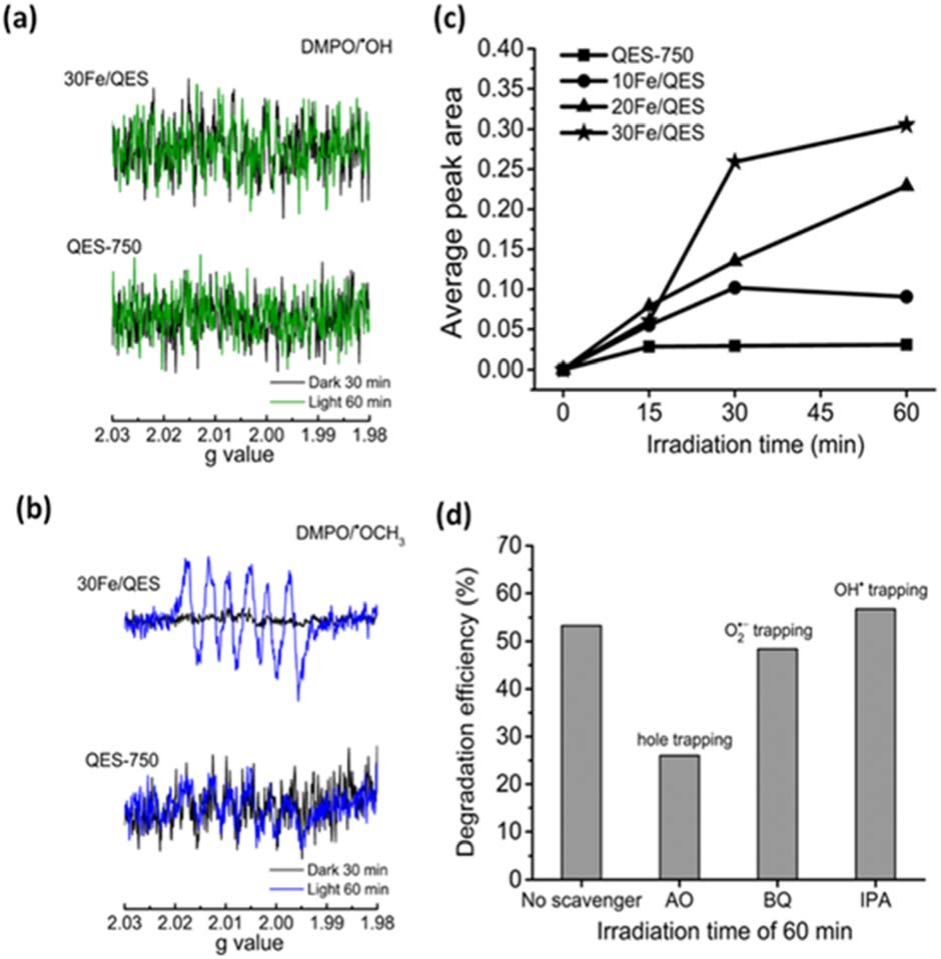
DMPO spin trapping ESR profiles used to identify the (a) hydroxyl radical (DMPO/˙OH) and (b) methoxy radical (DMPO/˙OCH_3_) under the photocatalytic degradation of 2-CP over QES-750 and 30Fe/QES. (c) Time-dependent average peak area obtained from the ESR signal (6 peaks) of DMPO/˙OCH_3_ under the photocatalysis system of QES-750 and Fe-modified QES, and (d) photocatalytic degradation efficiency of 2-CP by 30Fe/QES under UV-vis light irradiation of 60 min in the presence of scavengers (molar ratio of the scavenger: 2-CP = 2 : 1): ammonium oxalate (AO), benzoquinone (BQ), and isopropyl alcohol (IPA) were used to capture the photogenerated holes, O_2_˙^−^ and OH˙ radicals, respectively.

As shown in Fig. 8a, no ESR signals corresponding to the presence of the DMPO/˙OH adduct were detected in systems related to the QES-750 or 30Fe/QES samples. This is possibly due to the alkaline characteristic of the treated 2-CP solutions, which is in good agreement with previous works that reported lower concentrations of the DMPO/˙OH species in basic media.^65–67^ On the other hand, to evaluate the O_2_˙^−^ radical formation (as shown in Fig. 8b), six peaks were observed in 2-CP (aq) treated with QES-750 and 30Fe/QES under UV-vis light irradiation for 60 min. However, the presence of such peaks does not match with the ESR profile of the O_2_˙^−^ radical samples.^37–39^ The ESR signals with hyperfine coupling constants (g), *a*_N_ = 13.9, *a*_Hβ_ = 8.6 and *a*_Hγ_ = 1.6 agreed well with previous studies, indicating the formation of DMPO/˙OCH_3_ as the methanol molecules reacted with the photogenerated holes, giving the ˙OCH_3_ species (CH_3_OH + h^+^ → ˙OCH_3_ + H^+^).^38–40^ Consequently, the photogenerated holes can be detected by ESR in the form of the DMPO/˙OCH_3_ species. Methoxide are preferred on the basic catalyst surface. The absence of a typical DMPO/˙O_2_^−^ profile (e^−^ + O_2_ → O_2_˙^−^) could be possibly due to the weak signal and/or overlapping position with the ESR peak corresponding to DMPO/˙OCH_3_. Fig. 8c reports the values of the ESR peak areas corresponding to the density of the photogenerated hole in the treated 2-CP (aq) as a function of treatment times. From the results, the concentrations of the photogenerated holes in the treated 2-CP in decreasing order were obtained from the 30Fe/QES, 20Fe/QES, and 10Fe/QES treatments. A very low content of holes was generated in the QES750 treatments. Thus, Fe significantly improved the stability of the holes species, as well as boost the formation of holes in the photodegradation of 2-CP.

Next, scavenger testing, another complementary analysis, was conducted to explore the influences of possible reactive species (holes, O_2_˙^−^ and OH˙ radicals) on the 2-CP photocatalytic degradation. Comparative photocatalytic 2-CP degradation efficiencies with or without the addition of specific scavengers were measured after each solution was treated under UV-vis irradiation for 60 min. As shown in Fig. 8d, the significantly decreased 2-CP degradation efficiencies were obtained as ammonium oxalate (AO) was added into the system, giving only 26.0% compared with the control system (no scavenger, 53.3%). In addition, the degradation of 2-CP was slightly suppressed with the addition of benzoquinone for O_2_˙^−^ radical capture (48.4%). However, upon adding isopropyl alcohol (IPA) as the OH˙ scavenger, the degradation efficiencies were almost identical to that of the control system (56.8%). The scavenger testing results suggested that photogenerated holes played a major role. The O_2_˙^−^ radical had a minor influence on 2-CP degradation, whereas the OH˙ radical should not be responsible for the effective 2-CP degradation under the condition studied. This conclusion agreed well with the ESR results, as previously discussed.

**Fig. 9:**
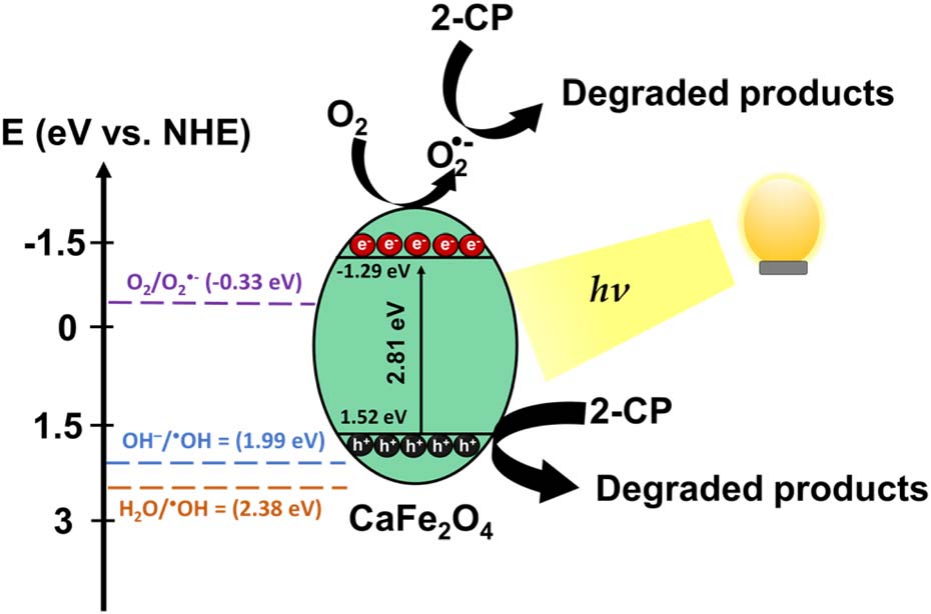
Proposed photocatalytic degradation of 2-CP over CaFe_2_O_4_ (30Fe/QES). According to the scavenging test, superoxide radicals and holes are important active species in 2-CP photodegradation.

The overall photocatalytic degradation processes of 2-CP over CaFe_2_O_4_ (30Fe/QES) are depicted in Fig. 9. Under light irradiation, the photogenerated electrons (e^−^) were excited from the valence band (VB) to the conduction band (CB), leaving holes (h^+^) at the VB of CaFe_2_O_4_. Based on the energy band structure of CaFe_2_O_4_, the hydroxy radicals could not be generated due the VB potential of CaFe_2_O_4_ (+1.52 eV) being less positive than +2.38 eV (˙OH/H_2_O, +2.38 eV *vs.* NHE).^63,68^ Alternatively, the photogenerated holes in VB can oxidize the 2-CP molecules. In addition, the CB potential (−1.29 eV) of CaFe_2_O_4_ was more negative than O_2_/O_2_˙^−^ (−0.33 V *vs.* NHE). The electrons in the CB can also reduce O_2_ to O_2_˙^−^, promoting the 2-CP degradation. From the energy band structure and scavenging study, it was noted that the photogenerated holes is a key radical active species for 2-CP degradation with the assistance of O_2_˙^−^ as a minor species.

Table 1 lists previous research studies that used spinel ferrite-based photocatalysts for chlorophenol degradation. However, a direct comparison of the photocatalytic performances obtained from this work and other studies is not simple, as all experiments were conducted using different treatment conditions (concentrations and structures of chlorophenols, light source, treatment times and catalyst loading). Table 1 gives the best efficiency obtained from each research group. Firstly, the reusable ferrite-based catalysts (reuse without the requirement of washing and heating steps) for chlorophenol degradation are calcium ferrite (produced in this work) and the ZnFe_2_O_4_ nanotube.^69^ Li *et al.* reported that under visible light irradiation, the ZnFe_2_O_4_ nanotube arrays derived from a sol–gel method provide a complete degradation of 4-chlorophenol (4-CP, 10 ppm) after 360 min treatments.^69^ The high photocatalytic performance of the ZnFe_2_O_4_ nanotube arrays was attributed to their narrow band gap (1.85 eV) and efficient channel for the photo-generated electron–hole pair over a well-oriented nanotube structure. However, because the amount of the ZnFe_2_O_4_ nanotube arrays catalyst was not clearly given, comparing the catalyst loading with this work is difficult. Despite being effective and reusable, the amount of 30Fe/QES required in the 2-CP degradation reactions was greater than previous works (Table 1). The low catalyst loadings (0.3–1 g L^−1^) were applicable to give the chlorophenol degradation efficiency of 70% and above for ferrite based-composites (Chitosan/CoFe_2_O_4_,^35^ CuFe_2_O_4_/TiO_2_,^70^ and Cu_0.5_Mn_0.5_Fe_2_O_4_/TiO_2_ ^70^). The effective separation of electron–hole separation under light irradiation and higher surface area (high density of catalytic active sites) resulted in high chlorophenol photodegradation efficiencies using ferrite-based nanocomposites.^70^ On the other hand, ZnFe_2_O_4_ ^36^ and CuFe_2_O_4_  ^70^ were ineffective in 4-CP degradation, and higher catalyst loading may be required to further improve the 4-CP degradation efficiency.

**Table 1:** Photocatalytic degradation efficiency of chlorophenols with spinel ferrite-based photocatalysts. N.R. = not reported

Material	Pollutant	Optimum conditions	Efficiency	Reusability/recyclability
ZnFe_2_O_4_ nanotube^69^	4-CP (10 ppm)	Catalyst coated on Ti foil	100%	Reusable
		Irradiation time: 360 min		5 Cycles (almost 100%)
		Light source: 500 W Xe lamp		
ZnFe_2_O_4_ ^36^	4-CP (30 ppm)	Catalyst loading: 0.75 g L^−1^	∼60% (pH 3)	N.R.
	Mixed with complexing agent	Light source: UV, 2.16 W, 18 mA, *λ* = 254 nm		
		Air = additional oxidant		
Chitosan/CoFe_2_O_4_ ^35^	2-CP (25 ppm, pH 10)	Catalyst loading: 1 g L^−1^	∼90%	Recyclable
		Irradiation time: 180 min		5 Cycles (80%)
		Light source: sunlight		Water washing followed by heating
CuFe_2_O_4_ ^70^	4-CP (200 ppm, pH 8)	Catalyst loading: 0.3 g L^−1^	∼65%	N.R.
		Irradiation time: 180 min		
		Light source: mercury vapor lamps (8 W) × 8		
CuFe_2_O_4_/TiO_2_ ^70^	4-CP (200 ppm, pH 8)	Catalyst loading: 0.3 g L^−1^	∼70%	N.R.
		Irradiation time: 180 min		
		Light source: mercury vapor lamps (8 W) × 8		
Cu_0.5_Mn_0.5_Fe_2_O_4_/TiO_2_ ^70^	4-CP (200 ppm, pH 8)	Catalyst loading: 0.3 g L^−1^	∼85%	Recyclable
		Irradiation time: 180 min		5 Cycles (>95%, irradiation time 270 min)
		Light source: mercury vapor lamps (8 W) × 8		Ethanol washing followed by heating
CaFe_2_O_4_ (30Fe/QES)	2-CP (25 ppm, pH ∼12)	Catalyst loading: 2.5 g L^−1^	86.1%	Reusable
This work		Irradiation time: 180 min		3 Cycles (70.5%)
		Light source: 300 W W lamp		

In comparison to prior findings, the materials in previous studies required longer treatment time (240–360 min) and the photocatalyst syntheses required expensive chemical raw materials.^35,69,70^ The advantages of using eggshell waste can be emphasized, as the Ca-rich waste derived CaFe_2_O_4_ materials showed photocatalytic activity that was comparable to that of other spinel-based systems, in addition to reusability without the requirement of surface regeneration (*e.g.*, washing, heat treatment and reactivation).

## Conclusions

Calcium ferrite (CaFe_2_O_4_, *E*_g_ = 2.81 eV) can be obtained, without CaO and Ca(OH)_2_ impurity phases detected, by using waste eggshell as a natural source. The green CaFe_2_O_4_ is applicable as a reusable photocatalyst in the oxidation of 2-chlorophenol (2-CP) under UV-vis light irradiation, achieving an 86.1% 2-CP removal efficiency. Photogenerated holes played the most important role in the radical pathway of 2-CP oxidation, compared with superoxide and hydroxyl radicals. Hydroxyl radicals were not detected in the reaction systems due to the band structure of calcium ferrite produced in this work and the alkaline conditions for 2-CP treatments. As eggshell waste is abundant and presumably renewable, manufacturing the ferrite catalyst on a larger scale should be feasible. From this work, the performance of eggshell-derived calcium ferrite should be further improved to lower the required catalyst loadings. Further research into potentially low-cost calcium ferrite-based composites may be carried out in order to achieve high-performance photocatalysts for various chemical-transformation applications. The development of such composites could have significant implications for environmental sustainability.

## Conflicts of interest

The authors declare no conflicts of interest.

## Supplementary Material
